# Unveiling fatty acid subtypes: immunometabolic interplay and therapeutic opportunities in gastric cancer

**DOI:** 10.3389/fonc.2025.1570873

**Published:** 2025-05-26

**Authors:** Huahuan Liu, Xin Hu, Xiangnan Zhang, Yanxin Yao, Liuxing Wu, Ye Tian, Hongji Dai, Kexin Chen, Ben Liu

**Affiliations:** ^1^ Department of Epidemiology and Biostatistics, Key Laboratory of Molecular Cancer Epidemiology, Key Laboratory of Prevention and Control of Human Major Diseases, Ministry of Education, National Clinical Research Center for Cancer, Tianjin Medical University Cancer Institute and Hospital, Tianjin Medical University, Tianjin, China; ^2^ Center for Single-Cell Omics and Tumor Liquid Biopsy, Zhongnan Hospital of Wuhan University, Wuhan, China

**Keywords:** gastric cancer, fatty acid metabolism, multi-omics technologies, immunotherapy, single-cell transcriptomics

## Abstract

**Background:**

The goal of this study was to develop a predictive signature using genes associated with fatty acid metabolism to evaluate the prognosis of individuals with gastric cancer (GC).

**Method:**

A total of 24 prognostic-related genes were identified by intersecting differentially expressed genes with 525 fatty acid metabolism (FAM) -related genes and applying a univariate Cox proportional hazards model. By performing consensus clustering of 24 genes associated with FAM, two distinct clusters of GC patients were identified. Subsequently, a risk model was constructed using 39 differentially expressed mRNAs from the two clusters through a random forest model and univariate Cox regression.

**Results:**

An R package, “GCFAMS”, was developed to assess GC patients’ prognosis based on FAM gene expression. The low-risk group exhibited a more favorable prognosis compared to the high-risk group across various datasets (P < 0.05). The model demonstrated strong predictive performance, with AUC values of 0.86, 0.623, and 0.508 for 5-year survival prediction in the training and two validation datasets. The high-risk group displayed lower IC50 values for embelin and imatinib, suggesting the potential efficacy of these drugs in this subgroup. Conversely, the low-risk group demonstrated an elevated response to immune checkpoints blockade therapy and a higher immunophenoscore, which was further validated in additional cancer cohorts. Public data from single-cell RNA sequencing confirmed that the characterized genes were predominantly expressed in endothelial cells and fibroblasts. Furthermore, the integration of transcriptomics and metabolomics revealed notable variations in fatty acid levels between the clusters, underscoring the clinical relevance of our fatty acid metabolism signature in shaping the metabolic profiles of GC patients.

**Conclusion:**

This developed FAM signature demonstrated potential as a biomarker for guiding treatment and predicting prognosis in GC.

## 1 Introduction

Gastric cancer (GC) is a common form of cancer worldwide, with nearly 1 million new cases and over half a million deaths reported in 2022 based on the most recent data from the World Health Organization International Agency for Research on Cancer (1). This places GC as the fifth most prevalent form of cancer and the fifth leading contributor to cancer-related deaths on a global scale. The risk factors contributing to developing of GC include infection by Helicobacter pylori, advanced age, high salt intake, and inappropriate dietary habits (2).

Lipids play a crucial role in the composition of cellular membranes and structural units of cells. In addition, lipids are also used for energy storage and metabolism and play essential roles as signaling molecules in various cell activities. Cancer is characterized by significant alterations in lipid metabolism, including fatty acids (FAs) and cholesterol (3). Cancer cells rely on lipid metabolism to obtain the energy, components for biological membranes, and signaling molecules needed for their growth, survival, spread, metastasis, and reaction to the tumor microenvironment and cancer therapy (4).

Malignant tumors primarily rely on *de novo* synthesis for necessary FAs, whereas normal cells typically obtain them through external sources (5–8). The increased production of saturated and monounsaturated FAs from *de novo* FA synthesis in cancer cells increase cell membrane saturation and resistance to chemotherapeutic drugs (9). Certain important enzymes involved in fatty acid synthesis, including ATP citrate lyase (ACLY), acetyl-CoA carboxylase (ACC), and fatty acid synthase (FASN), are upregulated in tumors and linked to aggressive tumor behavior and unfavorable prognosis (10–12). Moreover, the fatty acid transporter CD36, which is upregulated in cancer cells, facilitates the spread and resistance to treatment of tumor cells through increased absorption of long-chain FAs (13, 14).

FAs and lipid storage can also impact various types of immune cells, often leading to suppression of the immune system. Lipid buildup inside bone marrow cells promotes oxidative metabolism and supports immune-suppressing capabilities (15). The abnormal accumulation of lipids in tumor-infiltrating DCs (TIDCs) hinders their ability to present antigens (16). FA oxidation (FAO) is necessary to form CD8+ memory T cells (17). It is also crucial for differentiating Tregs and blocking FAO to avoid aggregation of immunosuppressive effector T-cell populations (18, 19). In conclusion, fatty acid metabolism (FAM) has impacts on immune cell function in the tumor microenvironment.

In recent years, the specific phenotype of abnormal FAM in tumor cells has gradually attracted great attention. Exploring the role of abnormal FAM in tumor biology and strategies to treat malignant tumors by targeting FAM pathways is receiving much attention. The role of FAM in GC in its clinical treatment is unknown and deserves further exploration. To evaluate the relationship between the FAM-related gene expression pattern and clinical outcomes of GC patients, 347 TCGA GC samples were collected and divided into two clusters. A FAM-related risk score was constructed to evaluate the prognosis of GC patients and assess the biological characteristics.

## 2 Materials and methods

### 2.1 Data acquisition

Gene expression patterns and detailed clinical information were acquired from The Cancer Genome Atlas database (TCGA), accessible through the Genomic Data Commons portal (GDC) (https://portal.gdc.cancer.gov/). Individuals within the dataset who did not have detailed survival records were not considered for further study. The training dataset consisted of a total of 347 clinical samples from GC (The Cancer Genome Atlas-Stomach Adenocarcinoma, TCGA-STAD). To validate our results, we included the GC dataset from the Gene Expression Omnibus (GEO) with the accession number GSE34942, consisting of 56 samples, and the Tianjin GC cohort, which served as validation sets and included 90 cases (20). Raw RNA-seq data from TCGA and Tianjin GC cohort were normalized to Transcripts Per Million (TPM) values. Microarray data from the GEO dataset (GSE34942, GSE13861, GSE15459, GSE26901, GSE26899, GSE28541) were normalized using the Robust Multi-array Average (RMA) method. To minimize potential batch effects across datasets, we applied the ComBat algorithm. Quality control measures were applied to remove low-quality samples and exclude those with incomplete survival data. Additionally, TCGA mutation data and copy number variation (CNV) data were extracted from the GDC, which hosts TCGA data alongside other genomic datasets.

### 2.2 Identification of the FAM clusters in GC

525 genes associated with FAM were compiled from the Gene Set Enrichment Analysis (GSEA) database. Differentially expressed genes (DEGs) between cancer and paracancerous tissue were identified by applying the criteria (21) |log2 Fold Change (FC)| > 1 and P < 0.05 using the R package ‘edgeR’, and then intersected with FAM-related genes. Afterwards, the univariate Cox proportional hazards model was used to identify 24 genes linked to the survival time of GC patients. The gene expression levels of 24 genes were used to uniformly categorize the GC samples into clusters. The ConsensusClusterPlus package (version 1.58.0) in R was employed to perform the consensus clustering algorithm, repeated 1000 times to ensure the stability of clusters (22). This process identified two clusters, labeled as “cluster1” and “cluster2”. Principal component analysis (PCA) confirmed the stability and reliability of the subtype classification. The operating system of the identified clusters was evaluated utilizing the Kaplan-Meier technique, with the log-rank test employed to examine any statistical disparities.

### 2.3 Conducting pathway enrichment analysis on genes that are expressed differently across clusters

To identify DEGs in two clusters, we used differential expression analysis based on an empirical Bayesian approach, which is implemented in the ‘limma’ package of the R language (23). DEGs were considered significant if their |log2FC| was greater than 1 and the adjusted P value was less than 0.05 (24). To adjust for multiple testing, we applied the Benjamini method and then conducted Kyoto Encyclopedia of Genes and Genomes (KEGG) and Gene Ontology (GO) enrichment analyses using the ‘clusterProfiler’ R package to investigate variations in biological processes between clusters. The findings were considered statistically significant, as the adjusted p-value was below 0.05.

### 2.4 Estimation of immune cell infiltration between clusters

The single-sample genome enrichment analysis (ssGSEA) algorithm (25) to perform a detailed analysis of 28 immune cell types in the tumor microenvironment. This analysis was based on specific gene panels defined in the literature for each immune cell subpopulation (26). To fully evaluate the immune status of cancer patients, we utilized the ‘estimate’ R package to determine the immune score, stroma score, and tumor purity. In addition, we employed the CIBERSORT, MCPcounter and TIMER algorithms to quantitatively assess the level of immune cell infiltration in the two clusters. The ssGSEA score indicated the proportional presence of different types of immune cells. Normalization of these scores to a unity distribution ensures that the minimum score is zero and the maximum is one, allowing for a standardized comparison across different immune cell types.

### 2.5 Changes in gene landscapes and mutation patterns in the two clusters are of great importance

Utilizing the GenVisR tool, version 1.26.0, within the R package, we identified the significantly mutated genes (SMGs). Afterward, two groups were analyzed for mutation patterns using MutationalPatterns version 3.4.0 and maftools version 2.10.0 from R packages. We then extracted the mutational signature from the GC dataset and performed a comparative analysis against the COSMIC V2 mutation database (https://cancer.sanger.ac.uk/cosmic), employing the cosine similarity method.

### 2.6 Construction and evaluation of the FAM-related risk signature

DEGs were selected to create a set of signature genes using the R software ‘limma’, based on the conditions of |log2FC| > 0.5 and P < 0.05 (27, 28). Volcano plots were used to demonstrate differential genes. The R package “randomForest” was used to identify key mRNAs, miRNAs, and lncRNAs contributing to the construction of the FAM signature. Model performance was optimized using 10-fold cross-validation, with 5 repeated runs to ensure stability and reduce overfitting. The mean error rate and cross-validation error were recorded for model selection. Genes significantly associated with survival risk were identified through univariate Cox regression analysis, and significant candidates (P < 0.05) were used to construct prognostic signatures. A multivariate Cox proportional hazards model was then applied to the selected genes, with the best model determined via stepwise regression. The model’s predictive ability was evaluated using the concordance index (C-index) and log-rank test. A risk score for each sample was computed using the coefficients derived from the multivariate Cox model. The correlation between risk scores and survival time was assessed using the Kaplan-Meier method and log-rank test. Additionally, the risk models were assessed using receiver operating curves (ROC). Multivariate Cox regression was used to assess the difference between this risk signature and traditional risk factors such as sex and age on the prognosis of GC patients.

### 2.7 Immunotherapy response prediction with FAM prognosis signature

Two groups undergoing immunotherapy were chosen to confirm the effectiveness of the FAM signature: one with advanced urothelial cancer (UC) treated with atezolizumab (IMvigor210 cohort, N = 298) (29) and the other with melanoma receiving anti-PD1 immunotherapy (Mela cohort, N = 121 (30). Data on clinical information and gene expression from the IMvigor210 cohort were obtained from the IMvigor210 dataset. Gene expression data of the anti-PD1 melanoma cohort were obtained from previous studies (30). Gene expression and prognosis data for the Mela cohorts (GSE78220, N = 26; GSE100797, N = 21) (31, 32) and the bladder cancer cohort (GSE176307, N = 90) (33), all treated with immunotherapy, were obtained from publicly available datasets.

### 2.8 Immunophenoscore analysis and chemotherapeutic response between different risk groups

The immunophenoscore (IPS), a highly effective molecular indicator of immune response, was employed for profiling the immune environments within tumors and cancer antigen profiles. Previous research involved gathering data on the weights of groupings of genes associated with the immune system, categorized into four main groups: major histocompatibility complex (MHC) molecules, suppressor cells, effector cells, and immune checkpoints or immunomodulatory factors. Weighted average Z-scores were computed using the gene expression levels. Additionally, we calculated the IPS by adding together the weighted average Z-scores of the four gene categories, resulting in a score between 0 and 10. The score measures the amount and behavior of immune cells in the tumor’s immune system, thus forecasting how the tumor will respond to immunotherapy. A higher IPS represents a higher immunotherapy response rate (26).

The study utilized the R package ‘pRRophetic’ to forecast the sensitivity of chemotherapeutic drugs in GC patients (34), determining IC50 through ridge regression and evaluating prediction accuracy with 10-fold cross-validation (35).

### 2.9 Cluster analysis of single-cell RNA-sequencing data

Data from individual cells (GSE167297) in five STAD samples underwent preprocessing and analysis using the ‘Seurat’ R package. To filter out low-quality cells, only cells with transcript counts between 300 and 10,000, detected in at least three cells per transcript, and with mitochondrial read fractions below 5% were retained for further analysis. For clustering, we employed the Louvain algorithm with a resolution parameter of 0.5, which was selected to effectively capture major cell populations while avoiding over-segmentation. This choice was validated through dimensionality reduction techniques (UMAP and t-SNE) and marker gene expression analysis, confirming that the clusters aligned with known major cell types. This resolution also aligned with commonly used thresholds in published scRNA-seq studies (36, 37). Primary cell categories were identified based on established cell markers obtained from published sources or the CellMarker database.

### 2.10 Study participants

A total of 42 individuals were included in the study, sourced from the Tumor Tissue Bank at Tianjin Cancer Hospital. Illumina NovaSeq 6000 was utilized for the RNA sequencing of every sample. METWARE performed untargeted metabolomics measurements in plasma. All the samples came from individuals diagnosed with GC with accurate histological and pathological assessments. All cases in the study were handled anonymously in compliance with legal and medical standards, as approved by the Ethics Committee of Tianjin Medical University Cancer Hospital and Institute, with informed consent obtained from all patients.

### 2.11 Collection and preparation of serum samples

Blood samples were obtained between 6:00 and 8:00 in the morning following a period of fasting to minimize the impact of food intake. Subsequently, all specimens were promptly placed in a freezer at -80°C. Prior to initiating the process, take out the samples from the -80°C freezer and allow them to thaw on ice until they are completely free of ice (all following steps should be carried out on ice). After thawing, vortexed the samples for 10 s and mix well. Pipetted 50 μL of the sample into a numbered centrifuge tube. Next, 300 microliters of an internal standard extract containing 20% acetonitrile and methanol in a 1:4 volume-to-volume ratio was mixed vigorously for 3 minutes, followed by centrifugation at 12000 rotations per minute for 10 minutes at 4°C. Following centrifugation, transfer 200 μL of the liquid above the sediment into a separate centrifuge tube with the same number, then store it in a freezer at -20°C for half an hour. After being spun at 12000 revolutions per minute for 3 minutes at 4 degrees Celsius, 180 microliters of the liquid above the sediment were transferred into a tube equipped with the appropriate injection vial for analysis using liquid chromatography-mass spectrometry. All sample extracts were mixed in equal parts to form a QC sample.

### 2.12 LC-MS analysis

#### 2.12.1 T3 UPLC conditions

The sample extracts were analyzed using an LC-ESI-MS/MS system (38–40) (UPLC, ExionLC AD, https://sciex.com.cn/; MS, QTRAP^®^ System, https://sciex.com/). The analysis parameters included the use of a UPLC column, specifically the Waters ACQUITY UPLC HSS T3 C18 (1.8 µm, 2.1 mm*100 mm), with the column temperature set at 40°C, a flow rate of 0.4 mL/min, and an injection volume of either 2 µL or 5 µL. The solvent system consisted of a mixture of water (0.1% formic acid) and acetonitrile (0.1% formic acid) with a gradient program starting at 95% acetonitrile and 5% water at 0 min, transitioning to 10% acetonitrile and 90% water at 10.0 min, maintaining that ratio until 11.0 min, then returning to 95% acetonitrile and 5% water at 11.1 min and staying at that ratio until 14.0 min.

#### 2.12.2 QTOF-MS/MS

The Triple TOF mass spectrometer was utilized for its capability to collect MS/MS spectra in an information-dependent manner (IDA) while conducting an LC/MS analysis. The TripleTOF 6600 acquisition software from AB SCIEX continuously assesses the complete scan survey MS data in this setting. It gathers and initiates the collection of MS/MS spectra based on predetermined conditions. During every iteration, 12 precursor ions with an intensity exceeding 100 were selected for fragmentation using a collision energy (CE) of 30 V, resulting in 12 MS/MS events with a product ion accumulation time of 50 msec each. The ESI source parameters were established with Ion source gas 1 and Ion source gas 2 set at 50 Psi each, Curtain gas at 25 Psi, source temperature at 500°C, and Ion Spray Voltage Floating (ISVF) at 5500 V or -4500 V in positive or negative modes, respectively.

Electrospray ionization quadrupole time-of-flight mass spectrometry.

Triple quadrupole (QQQ) and LIT scans were obtained using a QTRAP mass spectrometer, specifically the QTRAP^®^ LC-MS/MS System. This instrument is equipped with an ESI Turbo Ion-Spray interface and operates in both positive and negative ion mode. Analyst 1.6.3 software (Sciex) controls the system. The parameters for operating the ESI source were temperature of the source at 500°C; ion spray voltage at 5500 V (positive) and -4500 V (negative); gas I (GSI), gas II (GSII), and curtain gas (CUR) set at 50, 50, and 25.0 psi, respectively; high collision gas (CAD) was used. Calibration of the instruments was carried out using solutions of polypropylene glycol at concentrations of 10 and 100 μmol/L in QQQ and LIT modes, respectively. Each period was monitored for a distinct group of MRM transitions based on the metabolites that were eluted during that time frame.

### 2.13 Statistical analysis

Consensus clustering was utilized to perform clustering in order to identify robust structure across multiple clustering iterations (41). Survival curve for prognosis analysis was generated using the Kaplan-Meier method, with significance of differences determined by performing the log-rank test. Hazard ratios (HR) were calculated using univariate and multivariate Cox regression models, and their coefficients were displayed in forest plots. The Wilcoxon rank-sum test was used to compare continuous variables between two groups, while the chi-square test was employed for comparing classified variables. Spearman coefficients were applied to evaluate correlations among variables. Unsupervised clustering was performed to detect unique clusters based on the 39 genes’ expression in the signature of FAM. Orthogonal Partial Least Squares Discriminant Analysis (OPLS-DA) was utilized to confirm the efficacy of the clustering process (42). Chi-square tests were used to analyze the variations in baseline characteristics between cases and controls for categorical variables, while paired t-tests or Wilcoxon’s signed-rank tests were used for continuous variables. Significance was established with a P value less than 0.05, and all P values reported were calculated for both sides of the distribution. R 4.0.0 software was used for all statistical analyses.

## 3 Results

### 3.1 Development and validation of FAM clusters

**Figure 1:**
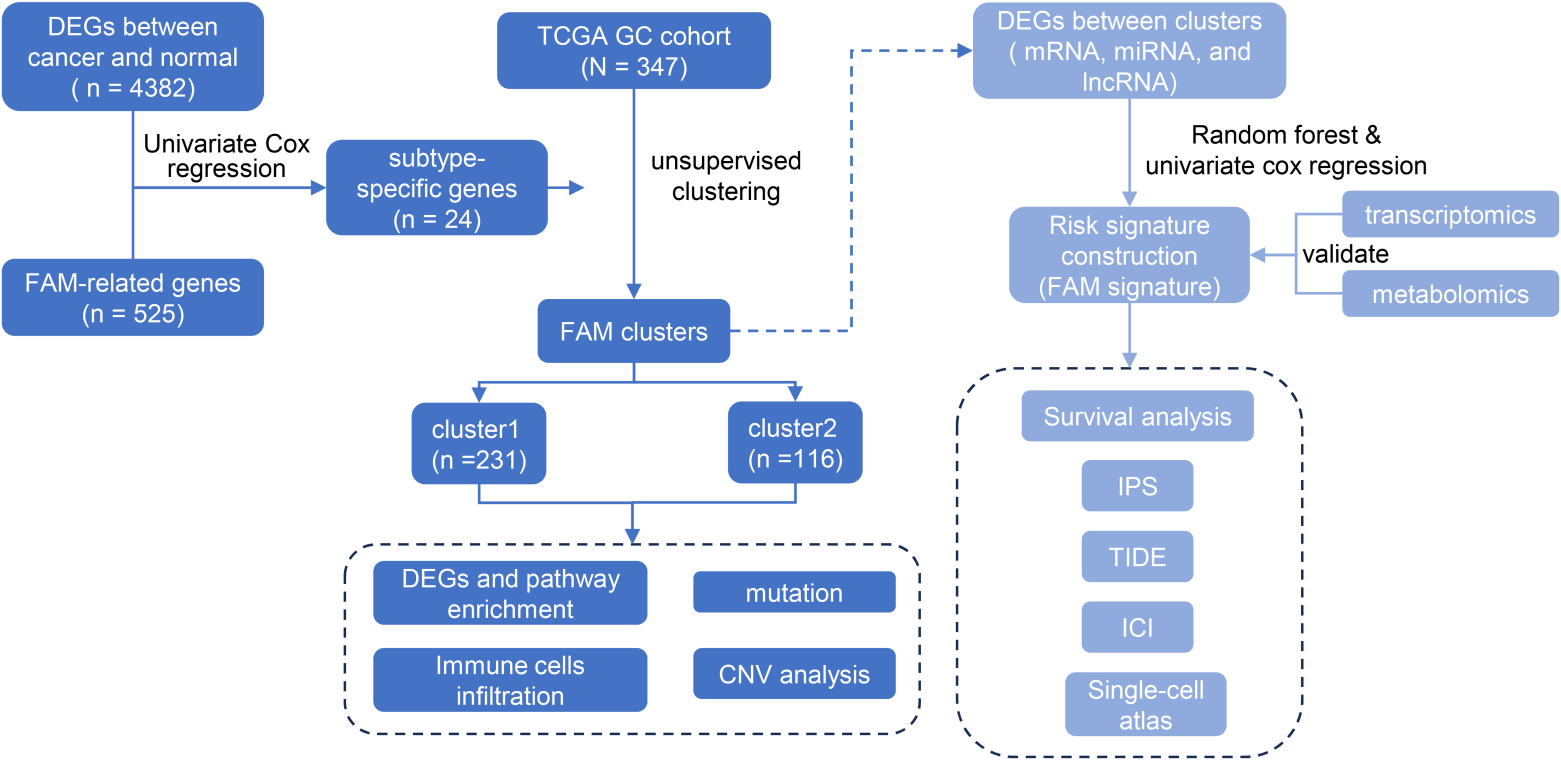
Flow chart of the experimental design and analysis of FAM related signature.

**Figure 2:**
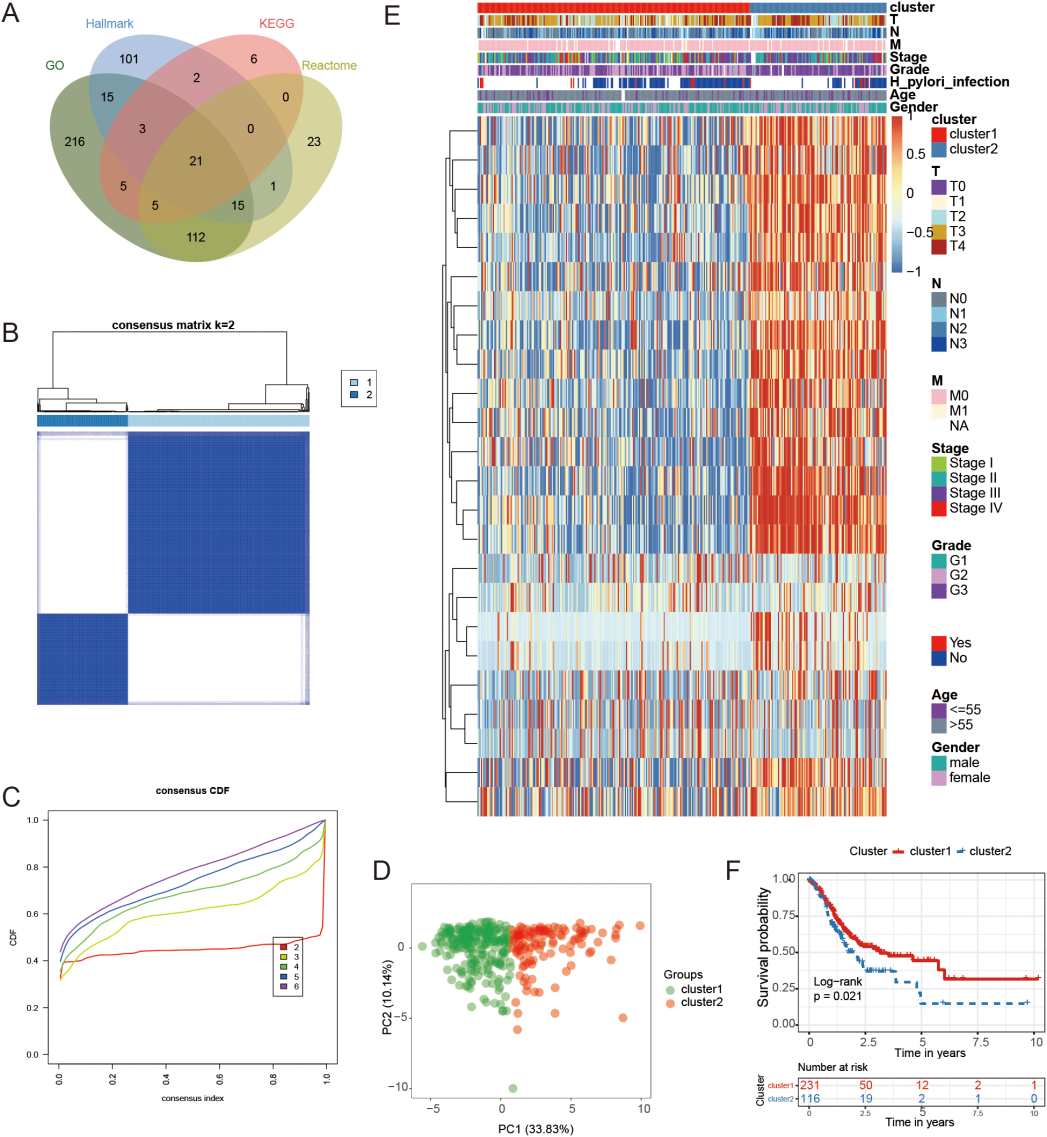
Classification and analysis of FAM subtypes. **(A)** The diagram illustrating the overlap of genes related to FAM from databases such as KEGG, GO, Hallmark, and Reactome. **(B, C)** The best classification effect was achieved by determining the optimal number of clusters (K=2) from CDF curves. **(D)** PCA was conducted on samples from GC using the principal component analysis method. **(E)** A heatmap illustrating the expression levels of 24 genes specific to each subtype. **(F)** Kaplan-Meier curves were used to forecast survival rates in individuals categorized into two clusters.

We devised a systematic flowchart to illustrate our study methodology (**Figure 1**). A total of 525 genes related to FAM were obtained from GO, Hallmark, KEGG, and Reactome databases (**Figure 2A**, **Supplementary Table S1**). After screening the DEGs in cancer and paracancerous tissues, intersected with 525 genes in FAM, and 24 prognostic-related genes were further screened using a univariate Cox proportional hazards model (**Supplementary Table S2**).

Subsequently, consensus clustering based on the expression patterns of the 24 FAM-related genes indicated that the most suitable number of clusters was two. This finding was confirmed by Cumulative Distribution Function (CDF) curves (**Figures 2B, C**). PCA revealed significant differences in gene expression between the two identified groups (**Figure 2D**). The expression patterns of the genes used for consensus clustering in the two clusters were visualized in **Figure 2E**, showing that cluster2 exhibited higher gene expression levels compared to cluster1.

Survival analysis revealed a significant difference in outcomes between the two clusters, as indicated by the log-rank test with a P-value of 0.021 (**Figure 2F**). Importantly, the observed prognostic differences were validated across three independent GEO GC datasets: GSE26899 (N = 93, P = 0.039), GSE26901 (N = 109, P = 0.0015), and GSE28541 (N = 40, P = 0.0011) (**Supplementary Figures S1A–C**). These findings demonstrated that GC samples could be classified into two groups based on 24 FAM-associated genes, revealing a distinct variation in prognosis between the groups.

### 3.2 Immune cell infiltration between FAM clusters

**Figure 3:**
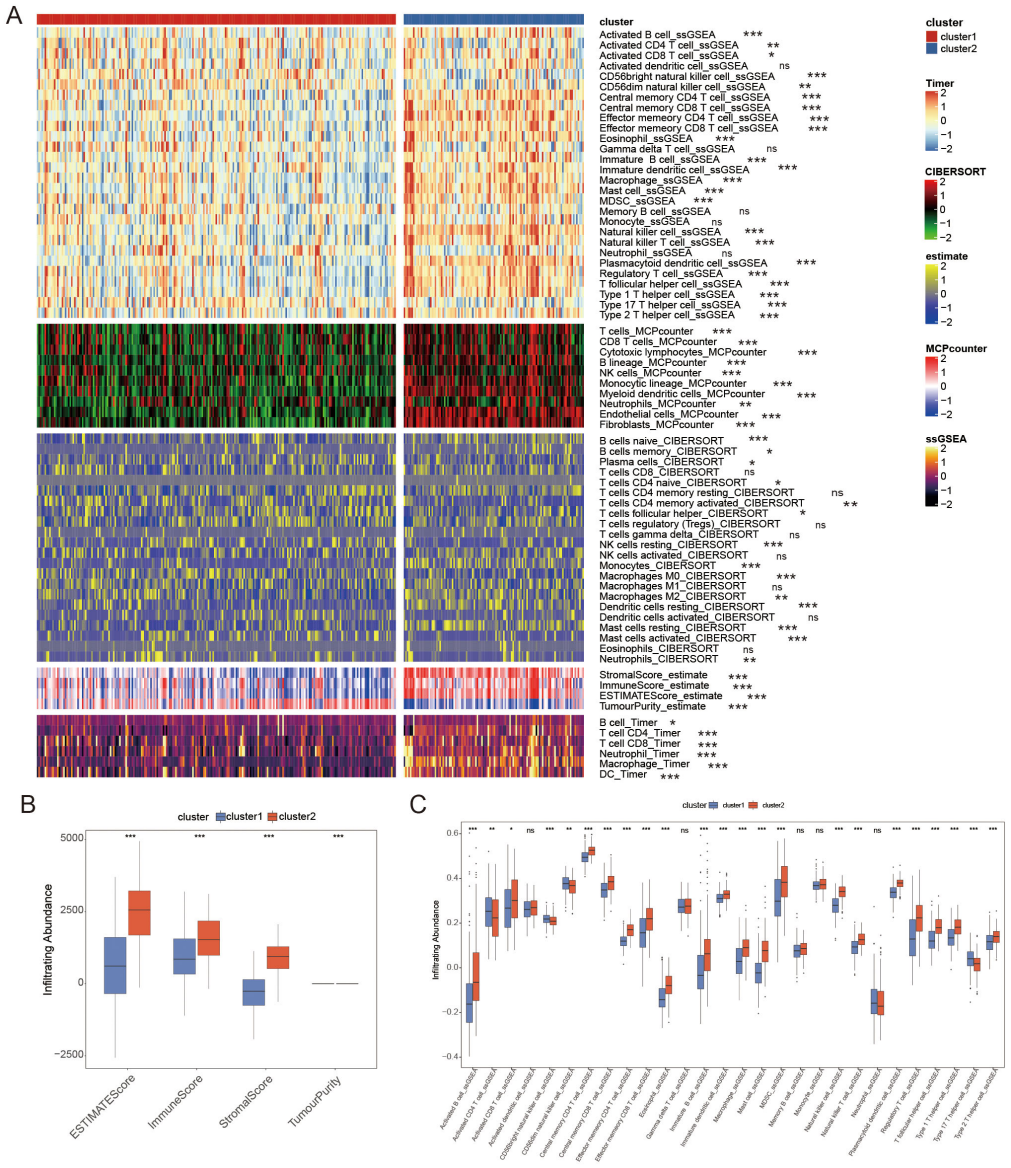
The distribution of immune cell infiltration as determined by four different algorithms. **(A)** Comparing the infiltration of immune cells in cluster1 and cluster2 using a Heatmap. **(B)** Tumor immune microenvironment scores between clusters. **(C)** There was a difference in the number of immune cells infiltrating between the two groups. * P < 0.05, ** P < 0.01, *** P < 0.001, ns P ≥ 0.05.

We evaluated the presence of immune cells in the two groups by analyzing the tumor immune environment through the immune score, stromal score, tumor purity score, and the abundance of 28 different immune cell types. A heatmap was used to illustrate the distribution of immune cell infiltration based on the four algorithms mentioned (**Figure 3A**). Compared to cluster2, which had a poorer prognosis, cluster1 exhibited a lower immune score and stromal score but a higher tumor purity score (**Figure 3B**). The degree of immune infiltration varied between the clusters, with cluster2 displaying more pronounced immune cell infiltration than cluster1 (**Figure 3C**).

### 3.3 Analysis of mutation patterns between FAM clusters

**Figure 4:**
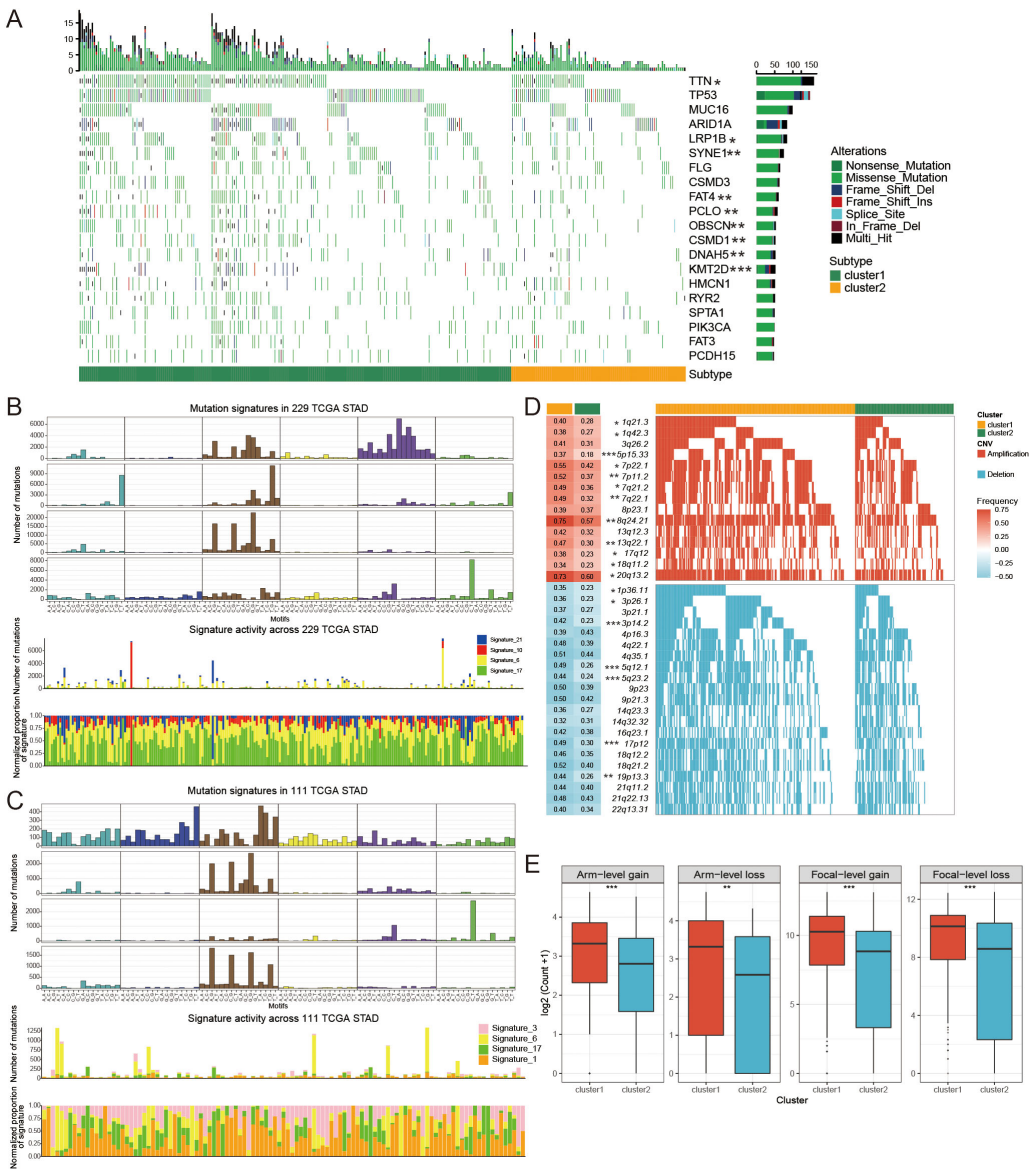
The mutational patterns and signatures of two FAM clusters. **(A)** Individuals in cluster1 and cluster2 created the graphical representation of tumor somatic mutations in the form of a waterfall plot. **(B, C)** Mutation signature identified in cluster1 **(B)** and cluster2 **(C)**. **(D)** Detailed charts showing copy number amplifications (increases) and copy number deletions (decreases) between different subtypes of FAM. **(E)** Distribution of specific and general changes in copy numbers between different subtypes of FAM. Significance levels were denoted as follows: *for P < 0.05, **for P < 0.01, ***for P < 0.001, and ns for non-significant results.

To investigate the relationship between FAM clusters and mutation patterns, we conducted SMG analysis. In our analysis of the top 20 mutated genes in GC, we found various mutated genes that differed between clusters, such as TTN, LRBP1, SYNE1, FAT4, and additional genes (**Figure 4A**). Four mutated signatures were derived from the COSMIC database by analyzing GC genomic somatic mutation data in order to explore differences in the mutational processes between the two subtypes. From the mutation data, four mutational patterns (signatures 6, 10, 17, and 21) were identified in cluster1, while cluster2 showed four different mutational patterns (signatures 3, 6, 17, and 1) as depicted in **Figures 4B, C**. Signatures 10 and 21 were unique in cluster1, and signatures 3 and 1 were distinctive in cluster2. The findings indicated that the mutation feature of cluster2 was linked to DNA damage and repair processes, like homologous recombination, resulting in the inability to repair DNA double-strand breaks.

We further investigated the differential somatic CNV alterations between FAM subtypes. CNV analysis identified 12 copy number gains, including 1q21.3 (P < 0.05), 5p15.33 (P < 0.001), 8q24.21 (P < 0.01) and 17q12 (P < 0.05) amplifications, and seven copy number losses, including 1p36.11 (P < 0.05), 3p14.2 (P < 0.05), and 5q12.1 (P < 0.001) (**Figure 4D**). The focal and arm level CNVs were compared through the GISTIC 2.0 approach. Cluster1 exhibited a greater overall load of copy number amplifications and deletions compared to cluster2 (**Figure 4E**).

### 3.4 Enrichment analysis between FAM clusters

**Figure 5:**
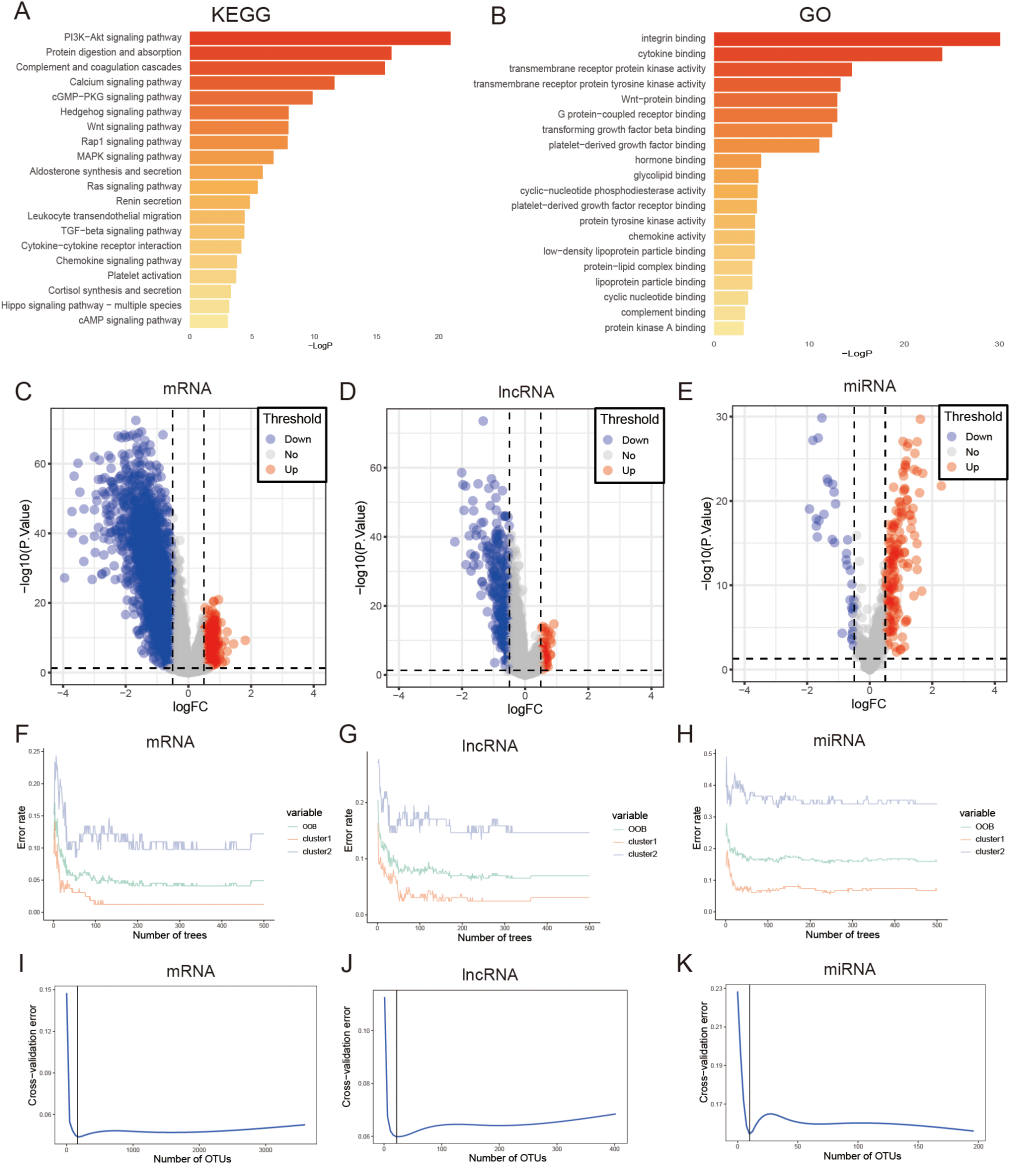
Performing enrichment analysis on DEGs between clusters of FAM using KEGG pathways. **(A)** and GO pathways **(B)**. **(C)** The volcano plot indicates that there were 3594 mRNAs that exhibited differential expression in the two subtypes of FAM. **(D)** The volcano plot indicates that there were 402 lncRNAs that exhibited differential expression in the two subtypes of FAM. **(E)** The volcano plot indicates that there were 196 miRNAs that showed differential expression in the two subtypes of FAM. **(F–H)** Error rate of the random forest model. **(I–K)** Cross-validation error of random forest model.

To further explore the effects of DEGs between the two clusters, we analyzed the signaling pathways of DEGs using KEGG and GO enrichment analysis. The findings indicated that the DEGs in the two groups were predominantly involved in various well-known signaling pathways such as the PI3K-Akt and MAPK pathways, as well as metabolic pathways, cell growth, and immune response (**Figures 5A, B**), and the PIA3K-Akt signaling pathway was associated with metabolism. All these results indicate that the two clusters exhibit differences in metabolism, immunity, and proliferation.

### 3.5 Development of a prognostic signature related to FAM

**Figure 6:**
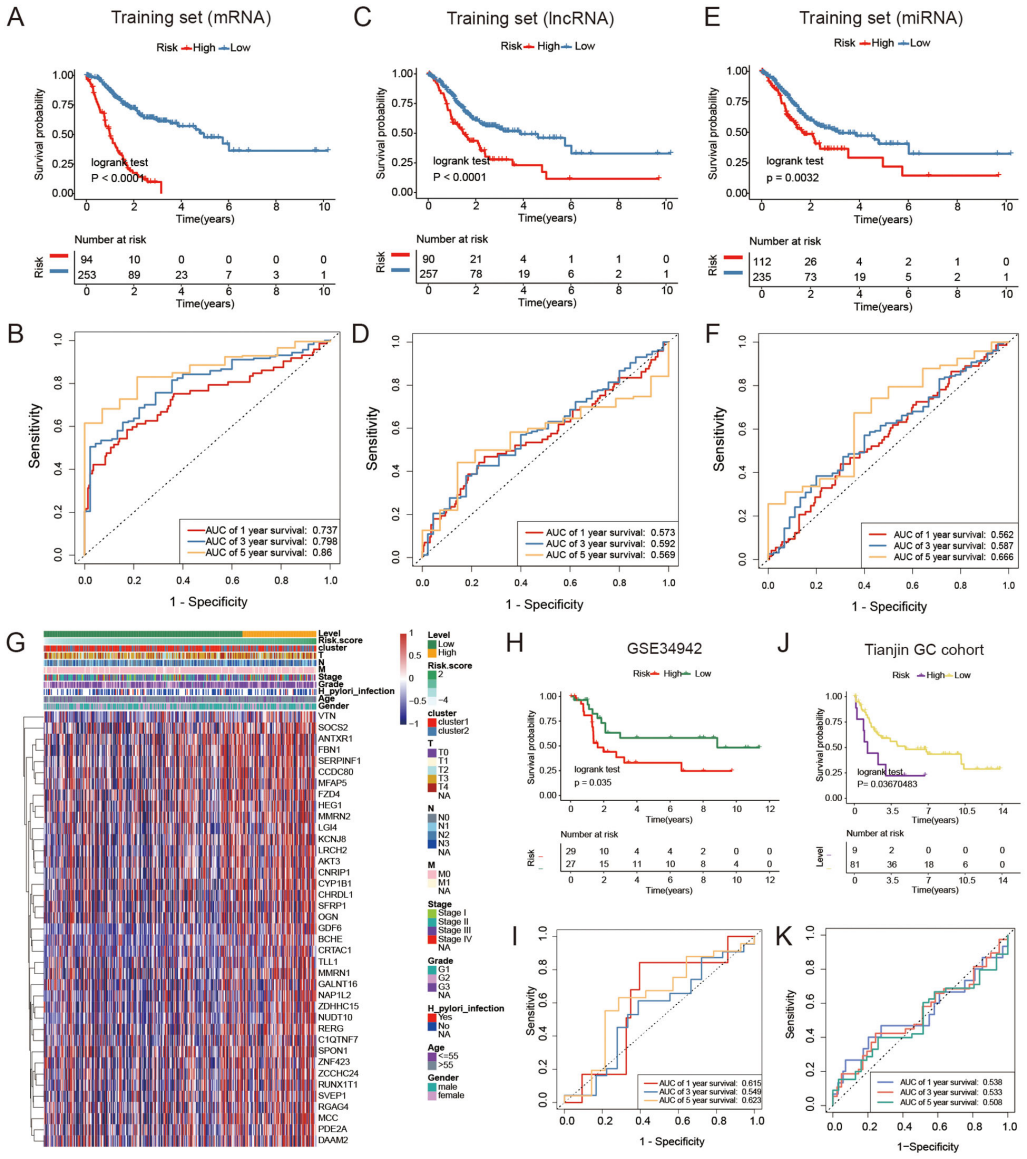
Comparison of different FAM risk models. **(A, C, E)** Kaplan-Meier plots were generated for GC patients based on their risk scores derived from a combination of mRNAs, lncRNAs, and miRNAs. Individuals classified as high-risk (red) had a lower overall survival rate (OS) compared to those classified as low-risk (blue). **(B, D, F)** ROC curves were generated to forecast the sensitivity and specificity of survival at 1-, 3-, and 5- years based on the risk scores derived from mRNA, lncRNA, and miRNA signatures. **(G)** The expression of mRNAs used to construct signature model in GC patients. **(H)** Kaplan-Meier analysis was performed on a group categorized as high- or low-risk based on 39 mRNAs in the GEO validation dataset (GSE34942, N = 56). **(I)** ROC curves to forecast the sensitivity and specificity of survival at 1-, 3-, and 5-years in the GEO validation dataset containing 56 subjects. **(J)** A Kaplan-Meier analysis was performed on the high-risk and low-risk groups created based on 39 mRNAs in the Tianjin GC dataset (N = 90). **(K)** ROC curves were utilized to forecast the sensitivity and specificity of survival at 1-, 3-, and 5- years in the Tianjin GC dataset (N = 90).

We screened for DEGs between the two clusters that could be used to construct a GC prognosis signature. First, we identified 3594 mRNAs, 402 lncRNAs, and 196 miRNAs differentially expressed between FAM clusters (**Figures 5C–E**, **Supplementary Table S3**).Then, the above genes were further screened, and 165 mRNAs, 22 lncRNAs, and 10 miRNAs were selected through the “randomForest” package (**Figures 5F–K**, **Supplementary Table S4**). Finally, 39 mRNAs, 4 lncRNAs, and 1 miRNA were further selected by univariate Cox regression analysis for GC prognosis signature construction (**Supplementary Table S5**). The cutoff values of high- and low-risk mRNAs, miRNAs, and lncRNAs were 0.414, 2.166, and 0.052, respectively, which were calculated by the R package “survminer”. Prognosis models built using mRNAs, lncRNAs, and miRNAs indicated a greater likelihood of survival for the low-risk group compared to the high-risk group (P < 0.0001, P < 0.0001, P = 0.0032; **Figures 6A, C, E**). The area under the curve values for the 5-year survival rates in the three categories were 0.860, 0.569, and 0.666, as shown in the **Figures 6B, D, F**, indicating that the 39 mRNAs constructed FAM risk signature could better predict the prognosis of GC patients. The heatmap showed the visualization of the expression level of the mRNAs in GC patient samples (**Figure 6G**).

The validation dataset (GSE34942) was utilized to confirm the accuracy of the GC prognosis signature constructed by 39 FAM-related mRNAs. The high-risk group, as determined by the risk signature in the TCGA GC cohort, had a worse outcome compared to the low-risk group (P = 0.035, **Figure 6H**). Nevertheless, the AUC values for 1-, 3-, and 5- years were lower compared to the training dataset (**Figure 6I**). The internal GC dataset we analyzed (Tianjin GC dataset, N = 90) showed that the low-risk group had a greater survival rate compared to the high-risk group (P = 0.037, **Figure 6J**). Furthermore, the AUC values were still lower than those in the training dataset (**Figure 6K**). Furthermore, to strengthen the generalizability of our risk signature, we included three additional external validation cohorts (GSE13861, GSE15459, GSE26901) (**Supplementary Figure S2**; P = 0.041, P =9e-04, P = 0.0027). The results further confirmed the prognostic utility of the 39-mRNA FAM risk signature in independent patient populations.

### 3.6 Assessment of the GC prognosis signature constructed by FAM-related mRNAs

**Figure 7:**
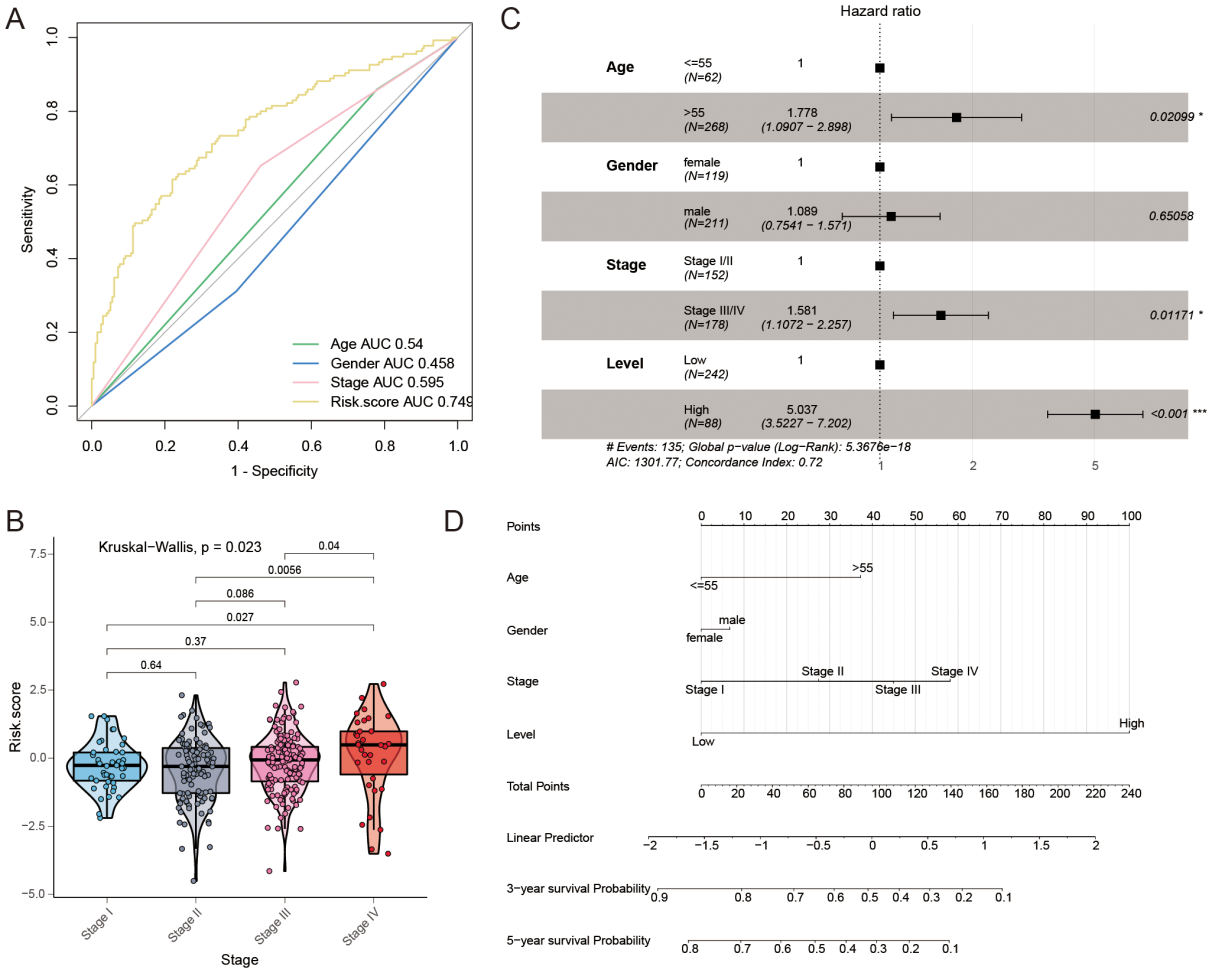
The predictive significance of the risk model for FAM. **(A)** ROC curves were used to evaluate the predictive ability of age, gender, stage, and risk score in determining sensitivity and specificity in GC patients, along with clinicopathological factors and a 39-mRNA signature-derived risk scores. **(B)** Comparing risk scores for FAM across various clinical stages. **(C)** Multivariate Cox regression was performed to analyze the relationship between clinicopathological factors and overall survival in patients with GC. **(D)** A nomogram was created utilizing the risk score for FAM along with established risk factors.

The AUC values of this constructed signature and common risk factors for GC were compared to further validate the validity of this signature. In the TCGA GC cohort, the risk score (AUC = 0.749) was more effective in predicting GC prognosis compared to traditional risk factors like pathological stage (AUC = 0.595), age (AUC = 0.540), and sex (AUC = 0.458) (**Figure 7A**), which suggested that the signature had a better prognostic ability for GC. Furthermore, there were differences in the risk scores of the four pathological stages in patients with GC (P = 0.023, **Figure 7B**). Multivariate Cox regression was employed to determine whether the signature has a prognostic value in GC independent of clinicopathological indicators such as age, pathological stage, and sex. In the multivariate Cox regression, the risk score had a hazard ratio (HR) of 5.037 with a 95% confidence interval (CI) of 3.523-7.202, showing statistical significance (P < 0.001, **Figure 7C**). A nomogram, created using a combination of the risk score and traditional risk factors, was utilized to forecast the chances of survival at 3- and 5- year for a patient with GC (**Figure 7D**). In addition, we developed an R package called “GCFAMS” (Gastric Cancer Fatty Acid Metabolism Score) for calculating the prognostic score of FAM in GC patients based on fatty acid metabolism gene expression (https://github.com/huxintmu/GCFAMS).

### 3.7 FAM-related mRNA signature predicts the response to immunotherapy and chemotherapy

**Figure 8:**
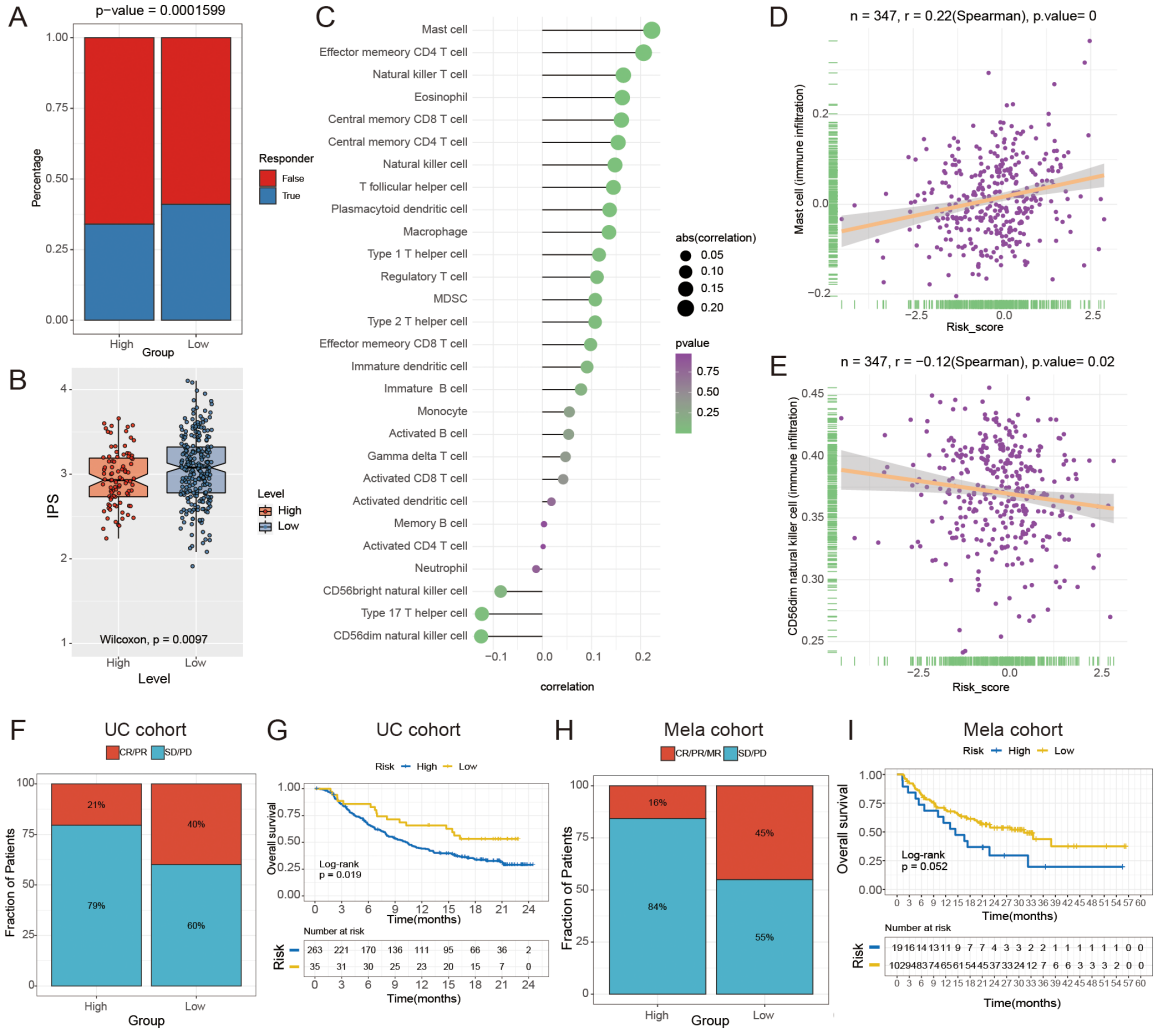
The relationship between the risk score for FAM and the effectiveness of immunotherapy. **(A)** The Tumor Immune Dysfunction and Exclusion (TIDE) analysis was used to forecast the response to immunotherapy in GC patients with high- and low- risk groups. **(B)** The boxplot indicates a notable contrast in IPS between the high- and low-risk groups (P = 0.0097). **(C)** The lollipop chart displays the correlation between the score of FAM and 28 different types of immune cells. **(D, E)** Spearman correlation between FAM score and mast cells **(D)** and activated CD56dim natural killer cells **(E)**. **(F, H)** Comparison of immune response to immunotherapy between high- and low- risk subgroups. CR indicates complete response, PR indicates partial response, SD indicates stable disease, and PD indicates progressive disease. **(G, I)** Kaplan-Meier analysis and the percentage of immune response to immunotherapy were compared between high- and low-risk groups in the UC **(G)** and Mela cohorts **(I)**.

We utilized the Tumor Immune Dysfunction and Exclusion (TIDE) analysis to investigate the potential of the FAM risk model in predicting immunotherapy response within the GC cohort (43). The findings indicated that individuals in the low-risk category exhibited a more favorable reaction to immunotherapy (**Figure 8A**). The findings indicated that the IPS was higher in the low-risk group compared to the high-risk group, as shown in **Figure 8B**. The study found that individuals with a low-risk score may have higher possibility of responding positively to immunotherapy. Next, we conducted a Spearman correlation analysis to investigate the connection between the FAM score and the infiltration of immune cells. As shown in **Figure 8C**, there was a relationship observed between the FAM risk score and immune cells.

Moreover, we found that the FAM score had the strongest positive correlation with mast cells (r = 0.220, P < 0.0001; **Figure 8D**) and a negative correlation with CD56dim natural killer cells (r = -0.12, P = 0.02; **Figure 8E**). In order to confirm the significance of the FAM score in immunotherapy, cohorts of malignant melanoma (Mela) and urothelial carcinoma (UC, the most prevalent form of bladder cancer) were utilized to investigate which individuals derive benefits from immunotherapy. Validation set results indicated that the low-risk group exhibited a greater response rate to immune-checkpoint blockade (ICB) in the Mela and UC cohorts (P < 0.05, **Figures 8F–I**). The findings indicated that individuals in the low-risk group were more likely to benefit from immunotherapy. To further confirm the robustness and real-world applicability of the FAM risk model, we performed external validation using an independent bladder cancer cohort (GSE176307) and two Mela cohorts (GSE78220, GSE100797). The results showed that low-risk patients had a significantly higher likelihood of responding to ICB (P = 0.033, P = 0.067, P = 0.032; **Supplementary Figure S3**), consistent with our findings in UC and Mela cohorts.

Furthermore, we evaluated the impact of chemotherapy drug reactions in both the high-risk and low-risk categories of the GC dataset. Chemotherapy drugs had distinct impacts on the high-risk and low-risk groups. In the high-risk group, embelin (P = 0.0023) and imatinib (P = 0.0048) showed lower IC50 values compared to the low-risk group (**Supplementary Figure S4**). The findings indicated variations in the effectiveness of immunotherapy and chemotherapy among different groups, with immunotherapy showing greater efficacy in the low-risk group and chemotherapy being more effective in the high-risk group.

### 3.8 Single-cell atlas distribution of genes that make up the signature for FAM

**Figure 9:**
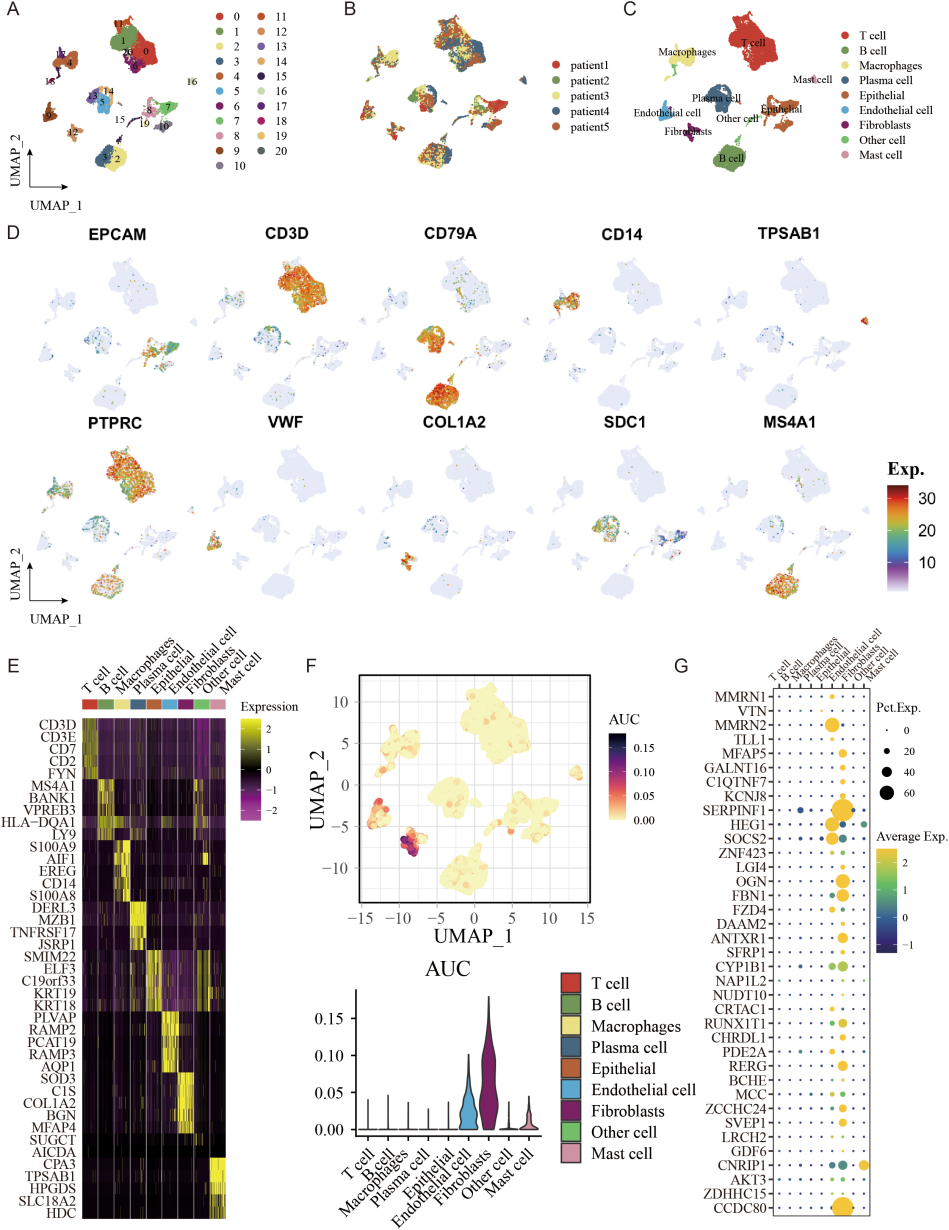
The single-cell atlas shows major cell lineages. **(A)** UMAP analysis was conducted to visualize and group 21 distinct cell clusters. **(B)** The distribution of cells with respect to the five patients (Patient1 to Patient5) is shown. **(C)** A UMAP plot displays nine primary lineages consisting of 23,060 cells. **(D)** UMAP feature plots were selected to display RNA expression of seven primary cell lineages. **(E)** Heatmap of the marker genes of the seven major cell lineages. **(F)** AUCell was used to analyze the gene enrichment of genes related to FAM that were used to create the signature. **(G)** FAM-related genes were utilized to create signatures in seven primary cell lineages.

The single cells of GC were clustered unsupervised by hypervariable genes, and the single-cell atlas comprised 21 clusters, as shown with a UMAP plot (**Figures 9A, B**). A single-cell atlas of multi-region GC cells included nine major cell populations (**Figure 9C**). T cells, B cells, macrophages, plasma cells, mast cells, epithelial cells, endothelial cells, fibroblasts, and others were included in the single-cell atlas. Detailed expression profiles and gene features of single gene markers are shown in **Figure 9D**. The expression heatmap of the marker genes of the major cell lineages is shown in **Figure 9E**. The gene enrichment analysis of FAM signature genes indicated that the genes were predominantly expressed in fibroblasts and endothelial cells, as shown in **Figures 9F, G**. Above all, single-cell analysis revealed that FAM signature genes were predominantly expressed in fibroblast cells and endothelial cells.

### 3.9 Joint analysis using transcriptomic and fatty acid metabolomic data to validate signature

**Figure 10:**
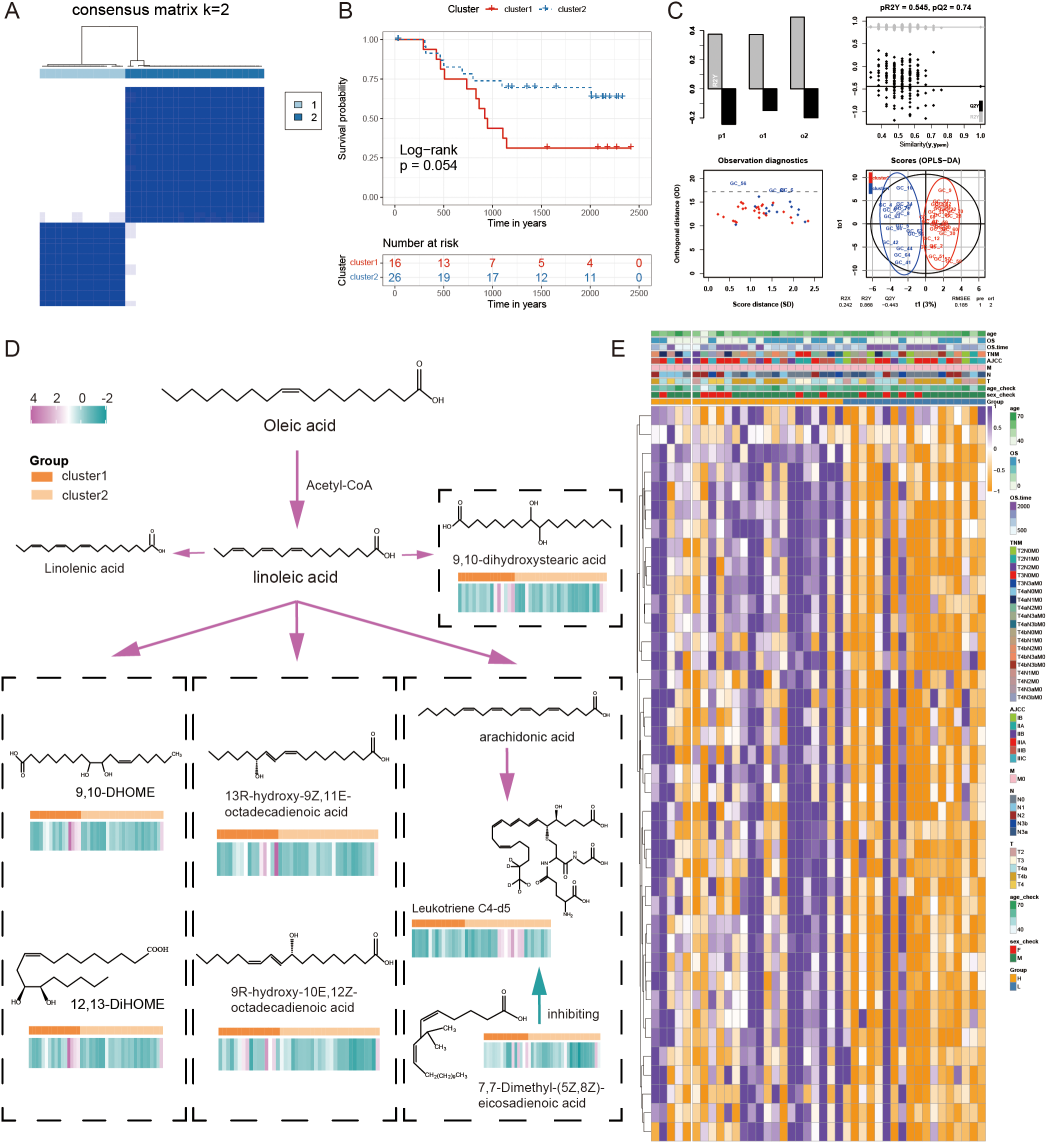
The combined examination of gene expression and metabolites in relation to the FAM pattern. **(A)** The ideal cluster number (K = 2) was identified based on the expression of signature genes related to FAM in 42 samples of GC. **(B)** Survival prediction of samples from two clusters was analyzed using Kaplan-Meier curves. **(C)** The OPLS-DA analysis of two clusters. **(D)** The differential FAM pathway diagram with content level differences. **(E)** A heatmap illustrating the levels of expression for 39 genes associated with FAM.

We enrolled 42 GC samples and performed a consensus clustering analysis based on FAM signature expression, showing the optimal number of clusters was two (cluster1 and clsuter2, **Figures 10A, E**), which was also defined by OPLS-DA analysis and CDF curves (**Figure 10C**, **Supplementary Figure S5**). Kaplan-Meier curves indicated that compared to cluster1, cluster2 had a better prognosis (log-rank test, P = 0.054, **Figure 10B**).

We identified the metabolites in the samples based on a broadly targeted metabolomic technique. A total of 1669 metabolites were detected using a non-targeted metabolomic technique for the detection of 42 mixed samples. Metabolomic variances among samples were explored through the utilization of a UPLC-MS/MS detection system, a custom-built database, and multivariate statistical techniques. Analysis of the variations between the two groups of metabolites revealed that a total of seven fatty acid-related metabolites exhibited statistically significant differences in expression, including ‘12,13-DiHOME’, ‘13R-hydroxy-9Z,11E-octadecadienoic acid’, ‘7,7-Dimethyl-(5Z,8Z)-eicosadienoic acid’, ‘9,10-DHOME’, ‘9,10-dihydroxystearic acid’, ‘9R-hydroxy-10E,12Z-octadecadienoic acid’, and ‘Leukotriene C4-d5’. In addition to Leukotriene C4-d5, other fatty acid-related metabolites showed higher levels in cluster1. The differential FAM pathway diagram was displayed with the content level differences in **Figure 10D**. The combined transcriptomic and metabolomic analysis of the same GC samples further revealed the importance of signature in FAM.

## 4 Discussion

Extensive evidence indicates that metabolic reprogramming plays a critical role in tumor progression (44). Tumor cells demonstrate increased FA production and uptake to support their needs, such as proliferation. A previous study utilized a FAM signature constructed from FAM-related genes to classify and evaluate the clinical therapies for colorectal cancer patients (45). In this study, a unique FAM profile was developed to personalize the evaluation of patients’ FAM levels, aiming to investigate how these levels influence the clinical outcomes and therapeutic strategies of GC patients.

To explore the clinical and biological implications of FAM in GC, we conducted unsupervised clustering based on FAM-related gene expression, categorizing the TCGA GC cohort into two distinct clusters. This stratification revealed a significant survival disparity between the two groups. Additionally, we identified altered genes and differences in immune cell infiltration between the two subgroups, and we performed a functional enrichment analysis of DEGs associated with the FAM subtypes. Subsequently, DEGs at different levels (mRNA, lncRNA, and miRNA) between the two clusters were identified for further analysis.

In the analysis of somatic mutations and CNVs, we identified several gene mutations and CNVs associated with FAM genes. Among them, LRP1B, a potential tumor suppressor gene (46), has been shown to enhance responses to immune checkpoint inhibitors (ICIs) in cancers with mutations in this gene (47). LRP1B mutation could potentially be used as a biomarker to anticipate the immune response and is linked to extended survival in melanoma and NSCLC immunotherapy groups (47). A comprehensive study across multiple cancer types demonstrated that patients with pathogenic or likely pathogenic LRP1B alterations experienced significantly improved outcomes with ICI treatment compared to those with alterations of unknown significance, regardless of their TMB/MSI status (48). Furthermore, research indicated that a solitary LRP1B mutation is associated with a poor response to ICI therapy and adverse outcomes in patients with HCC (49). In our study, cluster1 exhibited a higher LRP1B mutation rate compared to cluster2, suggesting that GC patients in cluster1 might achieve better outcomes with immunotherapy.

Additionally, SETDB1, located in the 1q21.3 region of human tumors, acts as an epigenetic barrier that suppresses the intrinsic immunity of tumors, making it a promising target for immunotherapy (50). Furthermore, a higher amplification of 8q24.21 was observed in cluster1 compared to cluster2. This region contains the C-MYC oncogene, which is associated with the development and progression of numerous types of cancers (51). Studies have shown that the activation of MYC signaling enables cancer cells to disrupt the surrounding microenvironment, allowing them to evade the body’s immune response (52). Amplification of 8q24.21 has been linked to tumor development and immune system modulation.

The ARID1A gene, located on 1p36.11, is the fourth most commonly mutated gene in GC. Han et al. demonstrated that ARID1A deficiency impairs fatty acid oxidation (FAO) by downregulating PPARα and altering the epigenetic landscape of specific metabolism-related genes (53). In our study, a higher deletion frequency of 1p36.11 was identified in cluster1 compared to cluster2. This finding suggests that patients in cluster2 may exhibit enhanced FAO capabilities.

PIK3CA initiates pathways that regulate cell growth, viability, division, movement, and morphology. It also promotes increased arachidonic acid metabolism through downstream mTORC2 signaling to sustain cell proliferation (54, 55). The PI3K-AKT-mTOR pathway plays a role in FAM in cancer, contributing to molecular heterogeneity and oncogenic signal transduction (44). The PI3Kδ enzyme complex is primarily found in the immune system, and its dysregulation-whether overactivation or insufficient activity-can lead to impaired and uncontrolled immune responses (56, 57). In this study, KEGG and GO analyses revealed that the two FAM clusters exhibited distinct differences in both FAM and immune activity.

Numerous studies have demonstrated a strong correlation between FAM and cancer progression, treatment, and immunity (58–60). FAs released by cancer cells influence immune cell infiltration within the tumor microenvironment. Disrupted lipid processing, such as upregulated FAO and *de novo* lipid synthesis, provides tumors with a competitive advantage against chemotherapy and radiation therapy, while also mitigating cellular stress associated with metastasis.

Additionally, T-cell activation requires *de novo* FA synthesis (61–63), and like other cell types, T cells rely on β-oxidation to degrade FAs as an energy source. FAO has been linked to various cell types, including CD8+ memory T cells and CD4+ regulatory T cells (19). Furthermore, the growth of B cells depends on monounsaturated FAs to sustain mitochondrial function and mTOR activity, thereby preventing excessive autophagy and endoplasmic reticulum stress (60).

In our research, cluster2 exhibited better survival outcomes compared to cluster1, potentially due to differences in immune cell infiltration. Previous study has also demonstrated that a high-fat diet increases FA uptake by cancer cells without significantly affecting tumor-infiltrating CD8+ T cells. This imbalance in FA distribution impairs the infiltration and function of CD8+ T cells, suggesting that optimizing metabolism could enhance tumor immunotherapy (64).

The observed AUC reduction in validation cohorts reflects real-world clinical complexity. Despite this, the model maintained significant survival stratification (P < 0.05) across all cohorts, demonstrating preserved clinical utility. Future multi-center studies with standardized protocols will further validate its robustness.

To assess the prognosis and effectiveness of immunotherapy and chemotherapy in patients with GC, we analyzed specific genes using various statistical methods, developed a signature consisting of 39 mRNAs, and confirmed its utility in guiding treatment decisions for immunotherapy and chemotherapy. Our study demonstrates that the FAM risk model is significantly associated with immune cell infiltration and immunotherapy response prediction. Specifically, the low-risk group exhibited a higher IPS and an improved response to immunotherapy, as validated in both GC and independent cohorts of Mela and UC. These findings suggest that the FAM score could serve as a potential biomarker to stratify patients for immunotherapy selection.

Moreover, we found that the FAM score was positively correlated with mast cells and negatively correlated with CD56dim natural killer cells, indicating that immune cell infiltration characteristics could influence treatment efficacy. Given that mast cells can promote an immunosuppressive tumor microenvironment (65, 66), while CD56dim NK cells play a critical role in tumor surveillance (67, 68), these correlations provide mechanistic insights into how the FAM signature may reflect tumor immune evasion strategies.

Furthermore, the integration of the FAM score with TIDE analysis revealed that patients in the low-risk category exhibited a higher likelihood of responding positively to ICB therapies (43), reinforcing the potential clinical utility of this model. By incorporating the FAM score into patient stratification strategies, clinicians may be able to better identify individuals who are most likely to benefit from immunotherapy, thereby improving personalized treatment approaches for GC.

The combination of transcriptome data with single-cell analysis provides a more comprehensive understanding of the mechanisms underlying cell heterogeneity in GC. A single-cell analysis was conducted to further investigate the expression of FAM-associated genes used in constructing the signature. Our results demonstrated that the genes incorporated into the signature were primarily expressed in endothelial cells and fibroblasts. Previous studies have suggested that fibroblasts contribute to the progression of GC (69), implying that they play a role in the malignancy of the cancer.

We performed an integrated transcriptomic and metabolomic analysis of the FAM signature to obtain multiple key differential fatty acid metabolites in the fatty acid metabolic pathway. Previous research has indicated that the imbalance in FAM processing can promote the proliferation of cancer cells, which usually exhibiting increased lipid storage compared to normal cells (70). An analysis using Mendelian randomization indicated that stearic acid was linked to a higher likelihood of developing colorectal cancer (71). In our study, cluster1 exhibited significantly higher levels of most fatty acid-related metabolites, except for Leukotriene C4-d5, and was associated with a relatively poorer prognosis compared to cluster2. The Leukotriene D4- Cysteinyl Leukotriene 2 receptor (CysLT2R) signaling pathway plays a key role in colorectal cancer, where CysLT2R has shown antitumor activity in intestinal epithelial cells. Since CysLT2R is a receptor for both Leukotriene C4 and Leukotriene D4, it is plausible that Leukotriene C4 may possess tumor-suppressive properties through its interaction with CysLT2R (72). The seven metabolites identified in our study are all metabolites of linoleic acid, which as a polyunsaturated fatty acid (PUFA) and an essential fatty acid, regulates cancer development by participating in a variety of *in vivo* metabolic pathways, including apoptosis, oxidative stress, and cell proliferation (73). A metabolomic investigation of hepatocellular carcinoma (HCC) revealed decreased levels of linoleic acid in portal vein and fecal samples of HCC patients compared to healthy controls (74). Further research has demonstrated that linoleic acid can stimulate CD8 T cells to boost their anti-tumor functions in both *in vivo* and *in vitro* settings (75). Furthermore, it was shown that intestinal-type GC could not produce arachidonic acid (AA) and adrenic acid (AdA) from linoleic acid, making GC cells immune to ferroptosis (76), indicating that polyunsaturated fatty acids might impact GC via the ferroptosis pathway. Therefore, various fatty acid-related metabolites can influence cancer development, progression, and therapeutic efficacy in various ways, while FAM signature, as a vital cancer biomarker, contributes to guiding the prediction of FAM levels in the human body.

Our study provided a comprehensive computational analysis of the FAM signature and its potential role in immune infiltration and therapeutic response. Using publicly available datasets and statistical models, we identified key genes associated with survival risk and validated the prognostic significance of the FAM-based risk score.

However, we acknowledge certain limitations in our study. First, our findings were based on bioinformatics analyses without direct experimental validation. Future studies should include *in vitro* and *in vivo* experiments to confirm the biological role of the identified genes. Second, prospective clinical validation is needed to confirm the clinical relevance of the FAM signature. One promising approach is the use of GC 3D models (77). GC organoids could be used to functionally validate the FAM signature, assess its impact on immune cell infiltration and test its predictive value for drug response. This would help bridge the gap between computational predictions and clinical applications.

Despite these limitations, our study provides a valuable framework for identifying prognostic biomarkers and generating hypotheses for future experimental research.

## 5 Conclusion

We developed a FAM signature to guide treatment and evaluate the prognosis of GC patients. Nevertheless, this study still has certain limitations. Further expansion of the sample size is required to confirm the findings of this research. Moreover, experiments still need to verify the relationship between FAM, immune cell infiltration, and outcomes of GC patients.

## Data Availability

This study analyzed datasets that were accessible to the public. The data involved in this article could be downloaded directly from TCGA and GEO datasets (GSE34942, GSE26899, GSE26901, GSE28541, GSE167297, GSE13861, GSE15459). Genome-wide microRNA and mRNA expression profiling of 90 Tianjin GC samples included a validation set of Tianjin GC data (20). For further information on this research, please contact the corresponding author.
