# Supplementary material for: Unveiling fatty acid subtypes: immunometabolic interplay and therapeutic opportunities in gastric cancer
Source: Front Oncol. 2025 May 26;15:1570873. doi: 10.3389/fonc.2025.1570873 (PMC12146350; doi:10.3389/fonc.2025.1570873)
Supplement: Supplementary file 1 [file DataSheet1.docx]

Supplementary Material

Unveiling Fatty Acid Subtypes: Immunometabolic Interplay and Therapeutic Opportunities in Gastric Cancer

Huahuan Liu^1, †^, Xin Hu^1,2, †^, Xiangnan Zhang^1, †^, Yanxin Yao^1^, Liuxing Wu^1^, Ye Tian^1^, Hongji Dai^1^, Kexin Chen^1, *^, Ben Liu^1, *^

**Affiliations:**

1. Department of Epidemiology and Biostatistics, Key Laboratory of Molecular Cancer Epidemiology, Tianjin, National Clinical Research Center for Cancer, Tianjin Medical University Cancer Institute and Hospital, Tianjin Medical University, Tianjin, 300060, China.

2. Center for Single-Cell Omics and Tumor Liquid Biopsy, Zhongnan Hospital of Wuhan University, 169 Donghu Road, Wuhan, 430071, China.

*****Correspondence to: Ben Liu; email: benliu100@tmu.edu.cn

Correspondence to: Kexin Chen; email: chenkexin@tmu.edu.cn

†These authors contributed equally to this work.

# Supplementary Figures and Tables

## Supplementary Figures


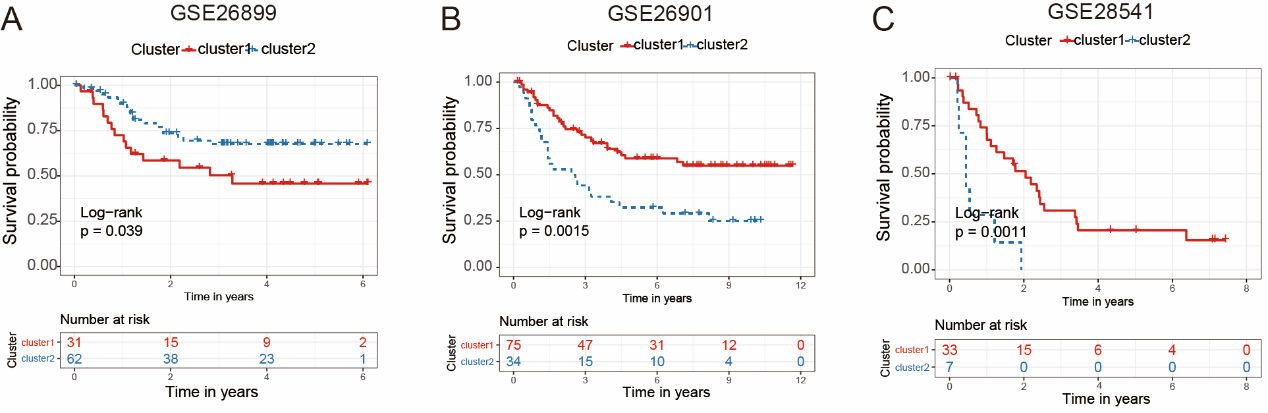


**Supplementary Figure S1.** Prognosis between the two clusters. (A-C) Comparison of prognosis between two clusters in TCGA GC datasets (GES26899 (N = 93), GES26901 (N = 109), and GES28541 (N = 40)).


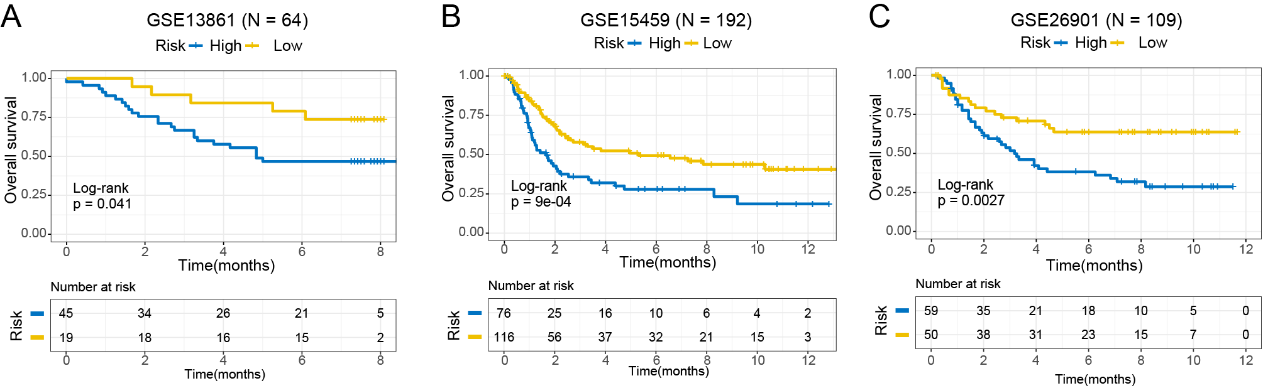


**Supplementary Figure S2.** A Kaplan-Meier analysis was performed on the high-risk and low-risk groups created based on 39 mRNAs in the additional GC cohorts (GSE13861, N = 64; GSE15459, N = 192; GSE26901, N = 109).


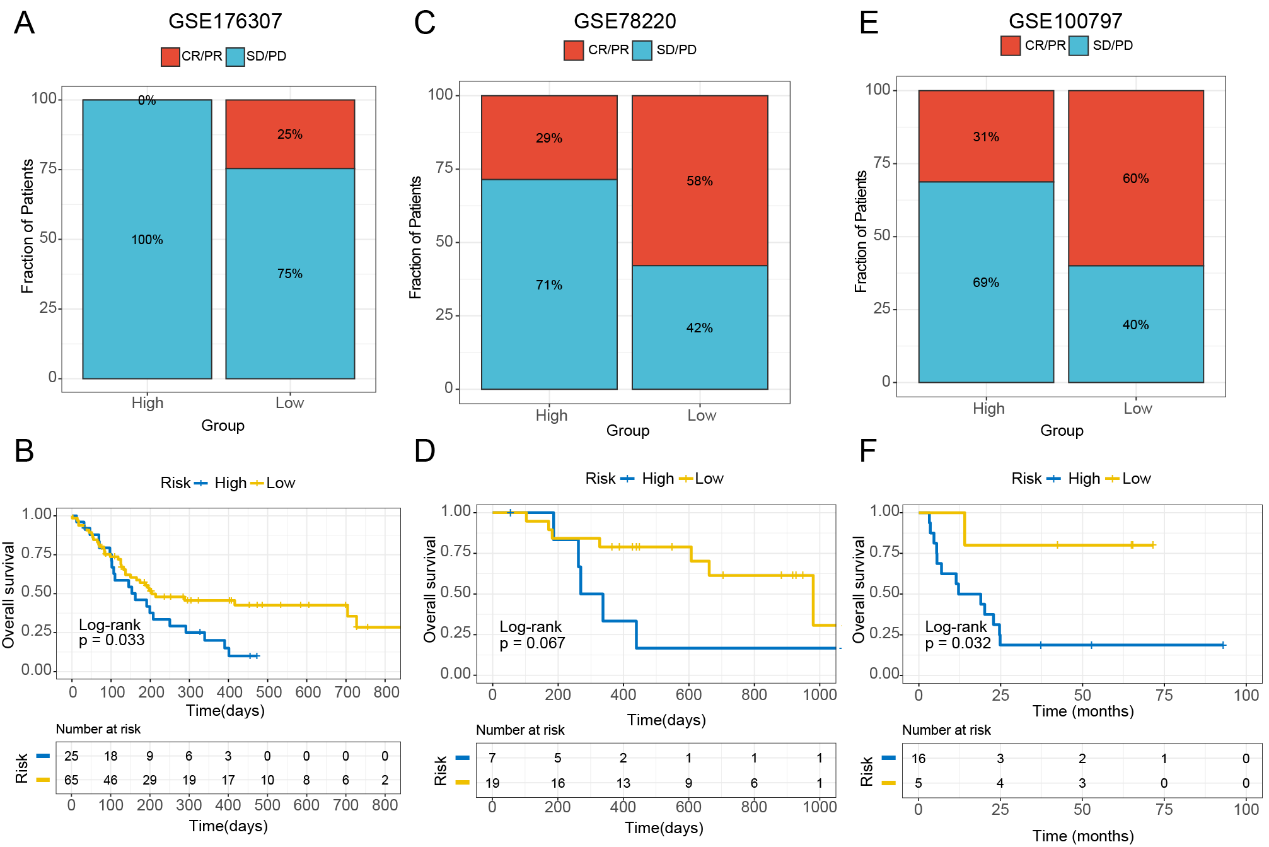


**Supplementary Figure S3.** Analysis of fatty acid metabolism subgroups in relation to immune checkpoint blockade therapy and survival outcomes. **(A, C, E)** Comparison of immune response to immunotherapy between high- and low- risk subgroups in bladder cancer (GSE176307) **(A)** and melanoma (GSE78220, GSE100797) **(C, E)** cohorts. CR indicates complete response, PR indicates partial response, SD indicates stable disease, and PD indicates progressive disease. **(B, D, F)** Kaplan-Meier analysis and the percentage of immune response to immunotherapy were compared between high- and low-risk groups in the bladder cancer (GSE176307) **(B)** and melanoma (GSE78220, GSE100797) **(D, F)** cohort**s**.


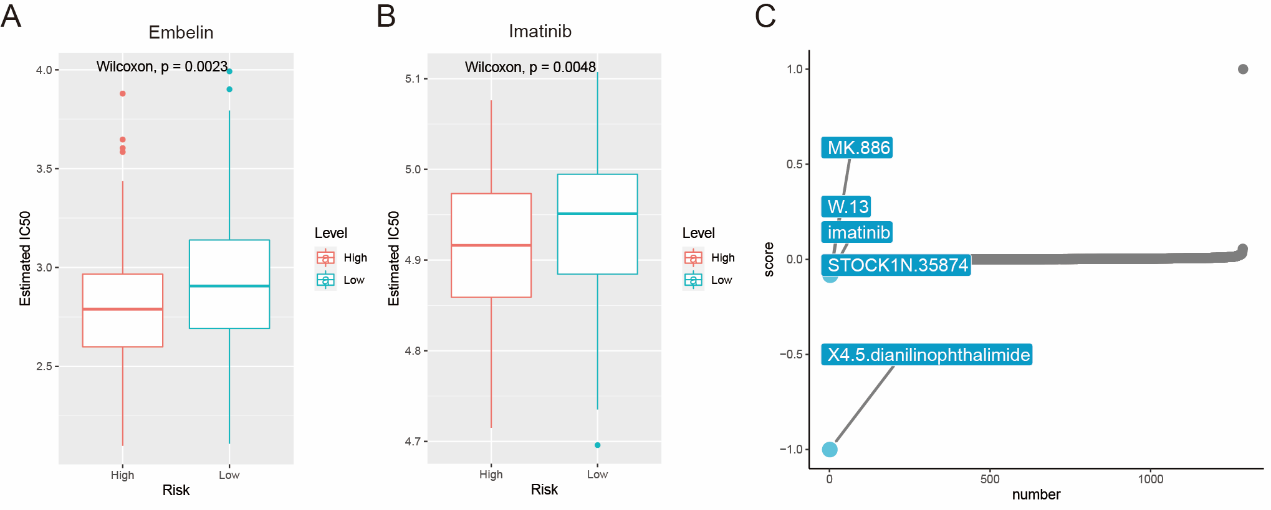


**Supplementary Figure S4.** The correlation between fatty acid metabolism risk score and chemotherapy. (**A, B**) The IC50s of embelin (**A**) and imatinib (**B**) in high-risk group were lower than that in low-risk group. (**C**) Small molecule targeted drugs for GC screened by Connectivity Map (CMap).


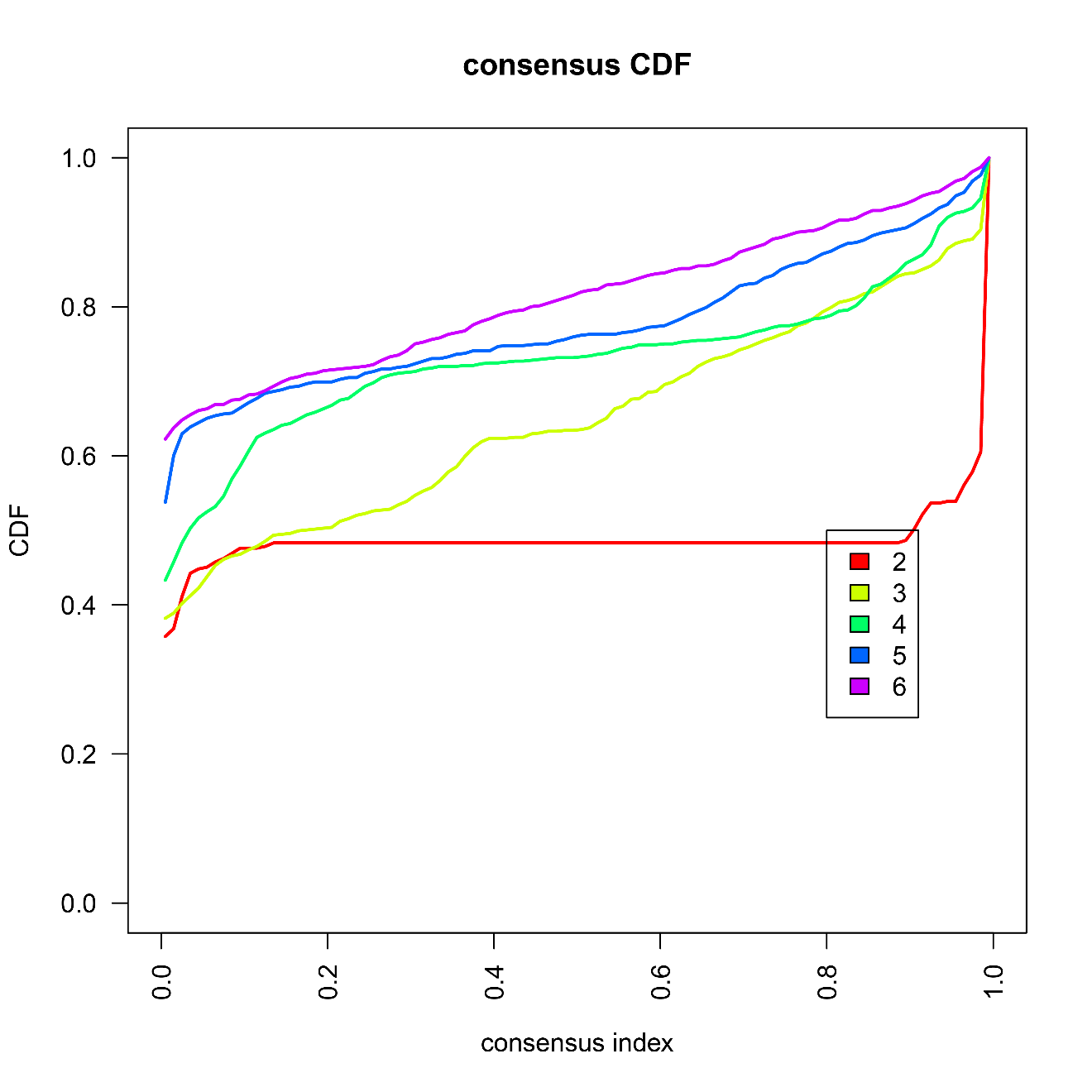


**Supplementary Figure S5.** The best classification effect was achieved by determining the optimal number of clusters (K = 2) from CDF curves.

**1.2 Supplementary Tables**

**Supplementary Table S1.** Fatty acid metabolism gene sets

**Supplementary Table S2**. Fatty acid metabolism related genes used to consensus cluster

**Supplementary Table S3**. The mRNAs, lncRNAs, and miRNAs differently expressed between clusters

**Supplementary Table S4**. The important mRNAs, lncRNAs, and miRNAs were screened using randomForest model

**Supplementary Table S5**. The mRNAs, lncRNAs, and miRNAs used to construct prognosis signatures
